# Genetically engineered T cells for the treatment of cancer

**DOI:** 10.1111/joim.12020

**Published:** 2013-01-19

**Authors:** M Essand, A S I Loskog

**Affiliations:** Department of Immunology, Genetics and Pathology, Uppsala UniversityUppsala, Sweden

**Keywords:** cancer, chimeric antigen receptor (CAR), clinical trials, genetic engineering, T cell receptor (TCR)

## Abstract

T-cell immunotherapy is a promising approach to treat disseminated cancer. However, it has been limited by the ability to isolate and expand T cells restricted to tumour-associated antigens. Using *ex vivo* gene transfer, T cells from patients can be genetically engineered to express a novel T cell receptor or chimeric antigen receptor to specifically recognize a tumour-associated antigen and thereby selectively kill tumour cells. Indeed, genetically engineered T cells have recently been successfully used for cancer treatment in a small number of patients. Here we review the recent progress in the field, and summarize the challenges that lie ahead and the strategies being used to overcome them.

## Introduction

Monoclonal antibodies (MAbs), such as trastuzumab (Herceptin) for the treatment of breast cancer, rituximab (MabThera) for B cell lymphomas and ipilimumab (Yervoy) for melanoma, have been successfully established during the last decade as anticancer drugs, and have rejuvenated the field of cancer immunotherapy 1. The potential of therapeutic T cells to traffic to sites of disease, expand and persist following a single injection remains a major advantage compared with MAbs. This has been well demonstrated through isolation, *ex vivo* expansion and adoptive transfer of tumour-infiltrating lymphocytes (TILs) for the treatment of malignant melanoma 2. However, T cell therapies for cancer have so far been limited by the lack of ability to isolate and expand high-affinity T cells restricted to tumour-associated antigens and by the limited *in vivo* expansion. By using gene transfer technologies, T cells can be genetically engineered to express a unique high-affinity T cell receptor (TCR) or a chimeric antigen receptor (CAR), both of which confer novel tumour antigen specificity. An adequate number of genetically engineered T cells can therefore be produced for adoptive transfer back to the patient. Indeed, genetically engineered T cells have recently been successfully used in cancer treatment 3–5. T cell therapy may have a clinical advantage compared with conventional therapies because of the specific lysis of antigen-positive cells, leaving other tissues intact.

**Fig 1:**
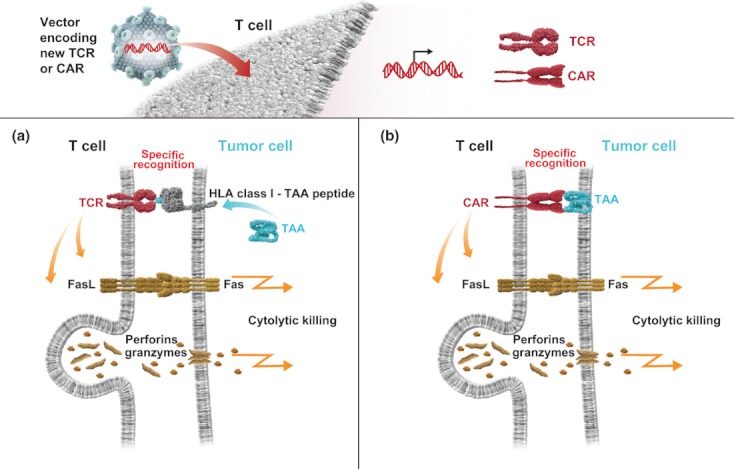
Specific antigen-recognition by a genetically engineered T cell leads to cytolytic killing of a tumour cell. The T cell is transduced with a viral vector encoding either a new antigen-specific TCR or chimeric antigen receptor CAR. (a) The tumour cell presents peptide fragments from tumour-associated antigen (TAA) on its surface in association with HLA class I. Specific recognition of the peptide/HLA complex leads to TCR signalling which triggers cytolytic killing of the tumour cell through secretion of perforins and granzymes and FasL-Fas interaction. (b) The tumour cell expresses a TAA on its surface. Specific recognition of the TAA leads to CAR signalling which triggers cytolytic killing of the tumour cell as described in (a).

The TCR is a heterodimer formed by the pairing of an alpha chain and a beta chain. The receptor interacts with an antigenic peptide presented by a major histocompatibility complex (MHC) molecule, in humans referred to as human leucocyte antigen (HLA), on the surface of a target cell for T cell-mediated cytolysis via induction of apoptosis in the target cell [Fig. 1(a)]. This is mediated by perforins, which insert themselves in the plasma membrane of target cells and form pores through which granzymes can enter and induce apoptosis of target cells. It is also mediated by Fas ligand, which induces apoptosis upon binding to its receptor Fas on target cells. The TCR is associated with the CD3 complex (gamma, delta, epsilon and zeta chains) and upon TCR recognition of an HLA/peptide complex the CD3 chains that contain immunotyrosine-activating motifs mediate signal transduction in the T cell. T cells equipped with a novel TCR can in theory target any protein antigen, including mutated intracellular antigens, which are often found in tumour cells, as they are processed and presented on the cell surface by HLA molecules. However, as the HLA is ‘polymorphic’, T cells with a novel TCR can only be used in a subset of patients. HLA-A2 is the most predominant HLA class I, present in ~50% of Caucasians. Consequently, most TCR gene transfer studies have focused on TCRs recognizing HLA-A2/peptide complexes. One disadvantage of TCR gene transfer is that tumour cells have a tendency to downregulate HLA class I expression during tumour progression and metastasis formation, which can render T cells inefficient.

A CAR, sometimes referred to as a T-body, chimeric immune receptor or chimeric artificial receptor, is a transmembrane molecule, which is composed of an extracellular binding domain derived from a single-chain antibody fragment (scFv) for recognition of a tumour-associated antigen and intracellular signalling domains for T cell activation. Hence, upon CAR binding to a tumour-associated antigen on the cell surface of a target cell, the CAR T cell will induce apoptosis in the target cell using the same mechanisms as ordinary T cells [Fig. 1(b)]. In contrast to a TCR, which recognizes a peptide fragment of an antigen presented by an HLA molecule on the surface of target cells, a CAR molecule recognizes an intact cell surface antigen, thus tumour cell recognition is HLA independent so there is no restriction in terms of patient selection. However, the requirement for the tumour-associated antigen to be a cell surface antigen excludes all mutated intracellular proteins from being targeted by CAR T cell-based therapy.

**Fig 2:**
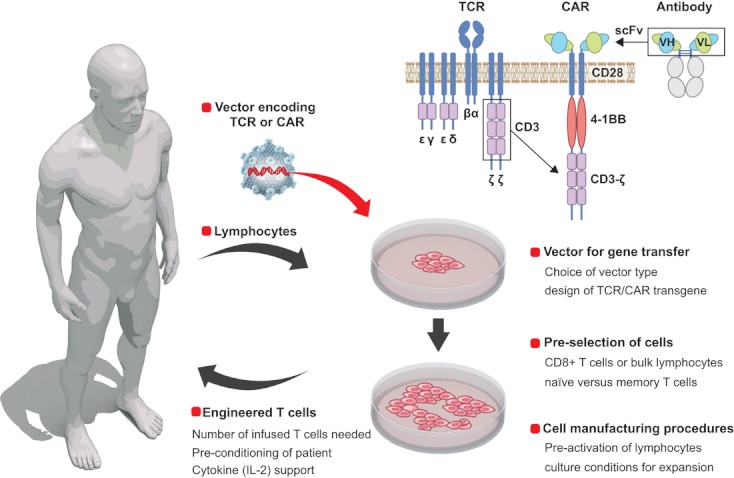
Genetic engineering and adoptive transfer of patient T cells. Lymphocytes are isolated from the peripheral blood of a cancer patient and transduced with a vector encoding either a new antigen-specific TCR or CAR. The engineered T cells are then expanded *ex vivo* before being adoptively transferred back to the patient. Important factors to consider during optimization of a clinical protocol are indicated.

T cells can be isolated from peripheral blood of cancer patients and genetically engineered with a new receptor before being transferred back to the patient. There are a number of factors that need to be considered for optimization of therapy, as shown in Fig. 2.

## TCR gene transfer to T lymphocytes

The first successful TCR gene transfer to human peripheral blood lymphocytes conferring antitumour reactivity was reported in 1999 using a TCR specific for an HLA-A2-restricted epitope of the MART-1 antigen, which is highly expressed by malignant melanomas 6. Since then, several studies have demonstrated that transfer of a tumour antigen-specific TCR into T cells yields an antigen-specific T cell population, including TCRs against an HLA-A2-restricted epitope of: the human MDM-2 oncoprotein 7, the gp-100 melanocyte differentiation antigen 8,9, the NY-ESO-1 cancer/testis antigen 10, the p53 tumour suppressor gene 11, carcinoembryonic antigen (CEA), which is reactivated by colorectal and other forms of cancer 12, the gp100 melanocyte differentiation antigen 13, the tyrosinase melanocyte differentiation antigen 14, the MAGE-A3 cancer/testis antigen 15, the MAGE-C2 cancer/testis antigen 16 and, most recently, the prostate and breast cancer antigen TARP 17. A TCR has also been cloned against an HLA-A24-restricted epitope of the Wilms' tumour 1 (WT1) antigen 18.

The first successful clinical trial with TCR gene-engineered T cells was reported by Morgan and colleagues in 2006, when infusion of autologous T cells with a TCR against an HLA-A2-restricted epitope of MART-1 yielded sustained objective clinical responses in two out of 15 patients (13%) with refractory metastatic melanoma 2006. This was followed by attempts to increase the effectiveness of T cell therapy by screening and isolation of highly active MART-1-reactive T cell clones 19 and by immunization of HLA-A2 transgenic mice, which have a non tolerant T cell repertoire, with peptides specific for the human gp100 melanocyte differentiation antigen. In a second reported TCR-engineered T cell therapy trial, 6 of 20 (30%) and 3 of 16 (19%) patients demonstrated clinical responses to MART-1 and gp100 respectively 13. Several studies using cloned TCRs are currently recruiting patients (summarized in Table 1).

**Table 1:** Clinical trials of the use of TCR-engineered T cells for cancer treatment

Trial no.	Status	Phase	Treatment	Pre-conditioning	Diagnosis	Sponsor
NCT01567891	Recruiting	I/II	MAGE HLA-A1 or NY-ESO-1 HLA-A2 TCR	No	Ovarian cancer	U-Penn
NCT01350401	Recruiting	I/II	MAGE HLA-A1 or NY-ESO-1 HLA-A2 TCR	Yes	Melanoma	U-Penn
NCT00704938	Terminated	II	p53 HLA-A2 TCR + IL-2	Yes	Kidney, melanoma, non-specific metastatic cancer	NCI
NCT00706992	Ongoing but not recruiting	II	MART-1 HLA-A2 TCR + peptide vaccine + IL-2	No	Melanoma	NCI
NCT00612222^a^	Terminated	II	MART-1 HLA-A2 TCR + peptide vaccine + IL-2	Yes	Melanoma	NCI
NCT00610311^a^	Terminated	II	gp100 HLA-A2 TCR + ALVAC vaccine + IL-2	Yes	Melanoma	NCI
NCT00923390	Recruiting	I/II	2G-1 (non-HLA restricted) TCR + IL-2	Yes	Metastatic renal cancer	NCI
NCT00910650	Recruiting	II	MART-1 HLA-A2 TCR + IL-2 + DC vaccine	Yes	Advanced melanoma	UCLA

Trials registered at clinicaltrials.gov as of 15 July 2012.

HLA, human leucocyte antigen; TCR, T cell receptor; DC, Dendritic cell; U-Penn, University of Pennsylvania; NCI, National Cancer Institute; UCLA, University of California Los Angeles.

a Terminated due to poor accrual.

### Increasing expression of the transferred TCR

A number of important factors should be considered for optimizing expression of the transferred TCR into T cells. First, codon optimization of the mRNA encoding the TCR has been found to significantly increase TCR expression levels 20,21. The insertion of a self-cleaving viral 2A peptide sequence between the alpha and beta chain, rather than having the two chains expressed independently or separated by an internal ribosome entry site has proven effective for achieving equimolar concentrations of the two chains 22. Furthermore, it has been demonstrated that endogenous CD3 expression, which needs to form a complex with the transferred TCR alpha and beta chains, is limiting for expression of the introduced TCR and that co-expression of the CD3 complex (gamma, delta, epsilon and zeta chains) increases the expression of the transferred TCR 23.

### Reduction of mispairing between exogenous and endogenous TCR alpha and beta chain molecules

Because a T cell already has a unique TCR, genetic transfer of a new TCR alpha and beta chain can lead to mispairing between an exogenous alpha and endogenous beta chain or *vice versa*. Mispairing gives rise to TCRs against unpredictable specificity and may generate receptors against self-antigens and thus cause autoreactive T cells. Furthermore, mispaired TCRs may compete for CD3 and thereby reduce the surface expression levels of the correctly paired transferred TCR. Several strategies have been used to avoid this potential problem. First, the constant domain of the TCR alpha and beta chain can be replaced with the murine domain. It was found that ‘murinized’ receptors were over-expressed on the surface of human lymphocytes compared with their human counterparts and were able to mediate higher levels of cytokine secretion when co-cultured with peptide-pulsed antigen-presenting cells. Preferential pairing of murine constant regions and improved CD3 stability seemed to underly these observations 24. However, murinized TCRs may evoke immune responses and potential clearance of transferred TCR-engineered T cells. Therefore, an alternative strategy is to introduce cysteine residues in the exogenous TCR alpha and beta chains at positions where they can interact and form disulphide bonds 25,26. This will lead to preferential pairing of the introduced chains. Alternatively, swapping position of two amino acids on the constant domains of the alpha and beta chains with naturally tight steric and electrostatic interactions can be employed as a ‘knob-in-hole’ approach. This favours selective assembly of the introduced alpha and beta chains, whereas mispairing would lead to unstable ‘knob–knob’ or ‘hole–hole’ interactions 27.

Efforts have been made to downregulate (knock-down) the endogenous TCR by small interfering RNA (siRNA) together with the transfer of a novel MAGE-A4-specific 28 or a WT1-specific TCR 29. Another elegant approach is to genetically knock-out the endogenous TCR by designer zinc-finger nucleases followed by transfer of a WT1-specific TCR 30. Both approaches are beneficial; they reduce/eliminate the risk of mispairing between exogenous and endogenous TCR alpha and beta chains and they reduce competition for CD3 molecules to form stable complexes of transferred TCR on the surface of T cells.

### Affinity optimization of the introduced TCR

Most human tumour-associated antigens targeted by TCR-engineered T cell therapy are also expressed, at lower density, in normal tissues. Therefore, autologous T cells recognizing these epitopes are normally of low affinity as all high-affinity clones have been deleted during thymic selection to prevent autoimmunity. Jakobsen and colleagues have in fact shown that TCRs from T cells that recognize self-tumour antigens have substantially lower affinities for cognate HLA/peptide complexes compared to their virus-specific counterparts 31.

One approach to isolate high-avidity T cell clones is to use HLA-A2 transgenic mice, which have not been exposed to the human tumour-associated antigen during thymic selection and therefore have a non tolerant T cell repertoire and the capacity to respond by generating T cell clones with high-avidity TCRs. Theobald and colleagues were the first to use this method to isolate a TCR against the human MDM-2 oncoprotein 7. It has also been used to isolate TCRs against p53 11, CEA 12, gp-100 13 and MAGE-A3 15. There is a potential risk that immunogenicity will form with elimination of TCR-engineered T cells when a TCR isolated from a mouse is used. This can be avoided by using transgenic mice for both human TCR and HLA genes 32.

A second successful approach is to isolate high-avidity HLA-A2-restricted T cell clones from an HLA-mismatched donor, thereby exploiting the natural repertoire of T cells from an HLA-A2-negative donor 33,34. However, this method can be cumbersome as allogeneic stimulator cells often yield T cells that respond to allogeneic epitopes not related to the HLA-A2-presented peptide.

A third possibility is to use HLA-A2 tetramers or other multimers composed of different peptides from tumour-associated antigens to select T cell clones with graft-versus-tumour reactivity from a polyclonal pool of graft-versus-host-disease T cells. In this way, a high-avidity clone against an HLA-A2-restricted epitope from PRAME was recently isolated 35.

Finally, *in vitro* affinity maturation of already characterized TCRs can be used. Yeast 36 or phage display 37 have been applied to express TCRs and select high-affinity TCRs through directed evolution. Furthermore, using a rapid RNA-based transfection system assay, single or dual amino acid changes in the CDR2 and CDR3 of a TCR were effectively introduced and mutants with significantly enhanced recognition of HLA-A2-restricted NY-ESO-1 and gp-100 peptides were identified 38. High-affinity TCRs can also be achieved through rational design using structural analysis to identify variation in a TCR that modulates antigen sensitivity 39.

### Soluble TCRs

Soluble TCRs have not only been developed for the purpose of crystallography but also as therapeutic reagents to mimic antibodies. A novel class of recombinant TCRs, termed ImmTACs (immune-mobilizing monoclonal TCRs against cancer), has recently been described. These receptors comprise a high-affinity soluble monoclonal TCR fused to a humanized CD3-specific scFv and can thereby redirect and activate naturally occurring T cells to lyse tumour cells 40. In addition, high-affinity TCR-like antibodies, which can be used both for therapy and as diagnostic tools, are currently being developed 41.

## CAR gene transfer to T lymphocytes

CARs are antibody-based extracellular receptor structures anchored into the cell membrane of T cells with a cytoplasmic domain mediating signal transduction. Eshhar and colleagues introduced the concept of CARs as early as 1989 42. Several groups have since confirmed the ability to redirect T cells using receptors encompassing different scFvs fused to the CD3 zeta or Fc receptor gamma (FcRγ) signalling domains. To date, CAR T cells have been reported to target a number of antigens on tumour cells including CD33 43, CD19 44,45, carboxy-anhydrase-IX 46, CD20 47, ERBB2-Her2/neu 48,49, GD2 50,51, PSMA 52,53, PSCA 54,55, mesothelin 56, CD171 57, VEGF-R2 58, MUC-16 59 and folate receptor-α 60,61. The ScFv portion of the CAR molecule is generally derived from a mouse MAb. This may evoke immune responses and potential clearance of CAR-engineered T cells. To avoid this possibility, fully human CARs can be constructed 62.

### First-generation CARs

The first-generation CAR molecules, with only an scFv against a cell surface antigen expressed on tumour cells and the cytoplasmic CD3 zeta chain signalling domain, were found to have limited clinical activity for the treatment of lymphoma 47, neuroblastoma 57, ovarian cancer 60 and renal cancer 46. First-generation CAR T cells demonstrated transient cell division and suboptimal cytokine production, and failed to produce prolonged T-cell expansion and sustained antitumour effects. This may not be surprising given that the signal through the TCR-CD3 zeta chain alone is insufficient for priming resting T cells 63.

### Second-generation CARs

Second-generation CARs were constructed to provide signalling both through the CD3 zeta chain and, primarily, the CD28 costimulatory molecule by placing the signalling domains in series as a single gene multidomain product 43,53. Constructs with the CD28 signalling domain proximal and the zeta chain distal to the membrane were found to be better expressed than constructs with the opposite orientation, and were capable of mediating up to 20 times more interleukin (IL)-2 production upon stimulation with solid-phase antigen compared with first-generation CARs 43. Subsequently, CAR constructs with costimulatory signalling domains from CD28, inducible costimulator (ICOS), OX-40 (CD134) or 4-1BB (CD137) in series with the CD3 zeta signalling region were evaluated using resting human primary T cells 64. It was found that second-generation CARs, providing any of these B7 or tumour necrosis factor receptor (TNFR) family costimulatory signals in series with CD3 zeta, confer self-sufficient antigen-driven clonal expansion and enhanced effector function in resting human T cells. Furthermore, addition of the CD28 signalling domain to CARs has been shown to enhance CAR T cell resistance to regulatory T cells 65.

It has been reported that CAR T cells with a 4-1BB signalling domain have improved *in vivo* persistence, tumour localization and antitumour activity 61 compared with CAR T cells with the CD28 signalling domain 5. Furthermore, a CAR with the CD27 signalling domain together with the CD3 zeta domain was recently evaluated. The greatest impact of CD27 was noted *in vivo*, where transferred CAR T cells with CD27 demonstrated heightened persistence after infusion, facilitating improved regression of human cancer in a xenogeneic allograft model 66. However, side-by-side comparisons of otherwise identical CAR T cells with either CD28, ICOS, OX-40, 4-1BB or CD27 signalling domains, in clinical trials under equivalent conditions, need to be performed before a general conclusion can be drawn as to which costimulatory domain is the most appropriate for CAR constructs.

### Third-generation CARs

Third-generation CARs have also been constructed containing CD3 zeta, CD28 and the OX-40 67 or the 4-1BB signalling domain 56. These receptors may provide a full complement of activation, proliferation and survival signals for enhanced antitumour activity. Despite encouraging preclinical results and some early clinical data, the use of third-generation CARs might have some disadvantages. One concern is that low avidity ‘off-target’ binding may trigger third-generation CARs with potent activation signals that can lead to a lethal ‘cytokine storm’. One patient treated with a third-generation CAR targeting Her2 died from adverse events due to Her2 expression in the lungs that led to excessive cytokine release and respiratory distress 49. In addition, third-generation CARs may reduce the signal threshold to a level at which the activation of grafted T cells can occur without triggering antigens. Signal leakage may be a problem for clinical applications of these CARs. Moreover, the exact amino acid sequence and order of the intracellular signalling domains are based on empirical findings, and the optimal CAR format for T-cell activation remains unclear.

### Ongoing clinical trials with CAR T cells

There are currently 36 trials of the use of CAR T cells for treatment of cancer registered at clinicaltrials.gov (Tables 2 and 3). Of these, only four trials have been completed, two are not yet recruiting patients and the remaining 30 trials are open for patient recruitment. Two thirds of the trials include patients with B cell leukaemia or lymphoma whilst the others are open to patients with non haematopoietic tumours. Approximately half of the trials are still investigating the use of first-generation CARs. However, to improve the likelihood of efficacy, the receptor is inserted into Epstein Barr virus (EBV)-specific T cells or co-expressed with a so-called transforming growth factor (TGF)-beta dominant negative receptor that blocks TGF-beta released into the tumour microenvironment. There are currently at least two registered trials of third-generation CARs, but only one is recruiting patients. The latter is targeting the EGFRvIII in patients with glioblastoma. The CAR used for targeting has both the CD28 and the 4-1BB signalling upstream of the CD3-zeta chain.

**Table 2:** Clinical trials of the use of CAR T cells for treatment of leukaemia and/or lymphoma

Trial no.	Status	Phase	Treatment	Pre-conditioning	Diagnosis	Sponsor
NCT00709033	Recruiting	I	CD19 CAR, EBV T cells	No	NHL, CLL	BCM
NCT00586391	Recruiting	I	CD19 CAR 1st vs. 2nd	No	NHL, CLL	BCM
NCT00608270	Recruiting	I	CD19 CAR 1st vs. 2nd 28	No	Relapsed or refractory NHL, CLL	BCM
NCT00840853	Recruiting	I/II	CD19 CAR, CMV, EBV and Ad trispecific T cells	No	ALL, CLL, NHL pre or post-HSCT	BCM
NCT01087294	Recruiting	I	CD19 CAR, allo-T cells	No	B cell malignancy relapsed post-HSCT	NCI
NCT00924326	Recruiting	I/II	CD19 CAR + IL-2	Yes	B cell malignancy	NCI
NCT01593696	Recruiting	I	CD19 CAR	No	Paediatric B cell malignancy	NCI
NCT01430390	Recruiting	I	CD19 CAR, alloEBV T cells	No	ALL post-HSCT	MSKCC
NCT01044069	Recruiting	I	CD19 CAR 2nd 28 vs. 4-1BB	No	ALL	MSKCC
NCT01029366	Recruiting	I	CD19 CAR 1st vs. 2nd 4-1BB	No	B cell malignancy	U-Penn
NCT00891215	Recruiting	I	CD19 CAR 1st vs. 2nd 4-1BB	Yes	B cell malignancy	U-Penn
NCT00968760	Recruiting	I	CD19 CAR ± IL-2	No	B cell malignancy post-HSCT	MDACC
NCT01497184	Recruiting	I	CD19 CAR	No	B cell malignancy post-alloHSCT	MDACC
NCT01318317	Recruiting	I/II	CD19 CAR, CM T cells	No	B cell malignancy post-HSCT	CHMC
NCT01475058	Recruiting	I/II	CD19 CAR, CMV + EBV bispecific, CM T cells	No	B cell malignancy post-HSCT	FHCRC
NCT01195480	Recruiting	I/II	CD19 CAR 1st, EBV T cells + EBV cell vaccine	No	B cell malignancy (paediatric) post-alloHSCT	UCL
NCT01316146	Recruiting	I	CD30 CAR 2nd	No	CD30^+^ NHL, HL	BCM
NCT01192464	Recruiting	I	CD30 CAR, EBV T cells	No	CD30^+^ NHL, HL	BCM
NCT00881920	Recruiting	I	Kappa light chain CAR 2nd	No	Kappa^+^ CLL, lymphoma or MM	BCM
NCT00621452	Ongoing but not recruiting	I	CD20 CAR 3rd and IL-2	Yes	B cell malignancy	FHCRC

Trials registered at clinicaltrials.gov as of 15 July 2012.

CAR, chimeric antigen receptor; EBV, Epstein Barr virus; CMV, cytomegalovirus; Ad, adenovirus; 1st, first generation; 2nd, second generation; 3rd, third generation; 28, CD28 domain; HSCT, haematopoietic stem cell transplantation; CM, central memory; NHL, non-Hodgkin's lymphoma; HL, Hodgkin's lymphoma; CLL, chronic lymphocytic leukaemia; MM, multiple myeloma; ALL, acute lymphoblastic leukaemia; BCM, Baylor College of Medicine; NCI, National Cancer Institute; MSKCC, Memorial Sloan-Kettering Cancer Center; U-Penn, University of Pennsylvania; MDACC, MD Anderson Cancer Center; CHMC, City of Hope Medical Center; FHCRC, Fred Hutchinson Cancer Research Center; UCL, University College London.

**Table 3:** Clinical trials of the use of CAR T cells for the treatment of non-haematopoietic tumours

Trial no.	Status	Phase	Treatment	Pre-conditioning	Diagnosis	Sponsor
NCT01109095	Recruiting	I/II	Her2 CAR, CMV T cells	No	Her2^+^ glioblastoma	BCM
NCT00889954	Recruiting	I	Her2 CAR, EBV T cells + TGFb DNR	No	Her2^+^ lung cancer	BCM
NCT00902044	Recruiting	I	Her2 2nd 28	No	Her2^+^ sarcoma	BCM
NCT00085930	Ongoing but not recruiting	I	GD2 CAR, EBV T cells	Yes/No	Neuroblastoma	BCM
NCT0064196	Recruiting	I	PSMA CAR	Yes	Prostate cancer	RWMC
NCT00673322	Recruiting	I	CEA CAR 2nd 28	No	Colorectal cancer	RWMC
NCT01373047^a^	Recruiting	I	CEA CAR 2nd 28	No	CEA^+^ liver metastases	RWMC
NCT00673829	Recruiting	I	CEA CAR 2nd 28 ± IL-2	No	Breast cancer	RWMC
NCT00004178	Completed	I	CEA CAR	No	Adenocarcinoma	RWMC
NCT00019136	Completed	I	Folate receptor CAR ± IL-2	No	Ovarian cancer	NCI
NCT01454596	Recruiting	I/II	EGFRvIII CAR 3rd 28 and 4-1BB ± IL-2	Yes	Glioblastoma	NCI
NCT00924287^b^	Terminated	I	Her2 CAR 3rd 28 and 4-1BB + IL-2	Yes	Metastasized Her2^+^ cancer	NCI
NCT01140373	Recruiting	I	PSMA CAR 2nd	Yes	Castrate metastatic prostate cancer	MSKCC
NCT00730613	Completed	I	IL13 zetakine CAR	No	Brain and CNS tumours	CHMC
NCT01460901	Recruiting	I	GD2 CAR multivirus specific	No	Post-allo HSCT neuroblastoma	CMHKC
NCT0000648	Completed	I	CE7R CAR 1st + IL-2	Yes	Neuroblastoma	FHCRC

Trials registered at clinicaltrials.gov as of 15 July 2012.

CAR, chimeric antigen receptor,; EBV, Epstein Barr virus; CMV, cytomegalovirus; DNR, dominant negative receptor; 1st, first generation; 2nd, second generation; 3rd, third generation; 28, CD28 domain; BCM, Baylor College of Medicine; NCI, National Cancer Institute; MSKCC, Memorial Sloan-Kettering Cancer Center; CHMC, City of Hope Medical Center; FHCRC, Fred Hutchinson Cancer Research Center; RWMC, Roger Williams Medical Center; CMHKC, Children's Mercy Hospital Kansas City. ^a^Delivered via hepatic artery; ^b^only one patient treated, with lethal outcome.

### CD19 CAR T cells

CD19 expression is restricted to normal and malignant B cells and therefore an appropriate target for CAR T cell therapy of B cell malignancies. Haematopoietic stem cells do not express CD19 and will therefore continuously produce new normal B cells. Nevertheless, an effective CAR therapy will eradicate existing normal B cells along with the malignant cells, but a transient loss of normal B cells will in most cases only cause manageable adverse events that can be treated by immunoglobulin-replacement therapy. Furthermore, CD19 expression is found on all tumour cells and is rarely lost during tumour cell progression. In a study conducted at Baylor College of Medicine, patients with B cell lymphomas were infused with first- and second-generation CD19 CAR T cells simultaneously. One CAR contained both CD28 and CD3 zeta, whereas the other contained CD3 zeta alone. The results of the study demonstrated that CD28 costimulation improves the *in vivo* expansion and persistence of CAR-engineered T cells 68. Rosenberg's group at the National Cancer Institute reported the results of the first patient to receive CD19 CAR T cells with both CD3 zeta and CD28 signalling. This patient was pre treated with lymphocyte-depleting chemotherapy before infusion of CD19 CAR T cells together with high-dose IL-2 69. After therapy, computed tomography scans revealed partial remission of the lymphoma, which lasted for 32 weeks. The main toxicity was the eradication of B-lineage cells from the bone marrow and blood. In a study conducted at the Memorial Sloan-Kettering Cancer Center 10 patients with chemotherapy-refractory chronic lymphocytic leukaemia (CLL) or relapsed B cell acute lymphoblastic leukaemia (ALL) were treated with CD19 CAR T cells containing both the CD28 and CD3 zeta signalling domains 70. The short-term persistence of infused T cells was enhanced by prior cyclophosphamide administration and was inversely proportional to the peripheral blood tumour burden.

Second-generation CD19 CARs, which include the cytoplasmic signalling domain of 4-1BB, have produced encouraging preclinical 71 and clinical 4,5 results. They exhibited enhanced antitumour activity and prolonged survival in a mouse model of primary human pre-B cell ALL and were significantly more effective than T cells expressing CD19 CARs containing CD3 zeta alone or CD28/CD3 zeta 71. In a small-scale clinical study conducted at the University of Pennsylvania (U-Penn), three patients with advanced chemotherapy-resistant B cell CLL (B-CLL) were treated resulting in two complete remissions and one long-lasting partial response 4,5. The CD19 CAR-engineered T cells expanded *in vivo* to a level that was more than 1 000 times higher than the initial engraftment level and persisted at high levels for 6 months in the blood and bone marrow and continued to express the CD19 CAR. Other than the tumour lysis syndrome, the only grade 3/4 toxic effects related to CAR T cells therapy were B cell aplasia, decreased numbers of plasma cells and hypogammaglobulinaemia. It is currently not fully understood why the results were so successful in this particular study. Differences in anti-CD19 scFv clones used and the fact that a lentiviral instead of a gamma-retroviral vector was used for gene transfer in the U-Penn study may have contributed to differences in the results. Furthermore, the method and length of T cell stimulation (CD3/CD28 magnetic beads vs. an agonistic CD3 antibody) before gene transfer and the handling of T cells post gene transfer may have contributed to the improved *in vivo* survival. Selection of patients and preconditioning regimens as well as the number of infused CAR T cells and cytokine support may also have contributed to the success. In the U-Penn study, preconditioning was performed, low numbers of T cells were infused, and patients did not receive IL-2 support.

Even if CD19 is an attractive target, nevertheless there have been efforts to further reduce on-target/off-tumour toxicity. For example, most low-grade lymphoma and B-CLL cells express monoclonal immunoglobulins carrying either kappa or lambda light chains. By targeting the kappa light chain of human immunoglobulin instead of CD19, a large proportion of normal B cells (all of which have lambda light chains) will be spared and consequently there will be reduced impairment of humoral immunity 72.

A method to develop universal allogeneic CAR T cells for therapy has been proposed in which the CD19 CAR is introduced by Sleeping Beauty transposons and the endogenous TCR alpha and beta chains are permanently knocked out by designer zinc-finger nucleases 73. As expected, using this method, it was found that these engineered T cells demonstrated redirected specificity for CD19 without responding to TCR stimulation. This represents a first step towards production of allogeneic T cells for transfer to B cell malignancies.

### CAR T cell therapy beyond the CD19 target

The encouraging results in the CD19 CAR T cell trials, especially in B-CLL, have stimulated expectations for therapy with genetically engineered T cells of nonhaematopoietic tumours. However, there are a number of differences that may make B-CLL and possibly other B cell malignancies more suitable targets for CAR T-cell therapy. First, B-CLL is an indolent disease whereas most solid tumours are fast growing. Secondly, B-CLL cells may form aggregates, but are seldom large or bulky. Therefore, CAR T cells may have better ‘access’ to B-CLL tumour cells than to tumour cells in bulky nonhaematopoietic tumours. Thirdly, B-CLL is derived from B cells, which are professional antigen-presenting cells (APCs) and may therefore provide better costimulation to CAR T cells. This may mean that CD19 CAR T cells will enable survival signals, besides CAR signalling, to persist longer than CAR T cells targeting non-APC tumours. Finally, CD19 CAR T cells will not only eliminate malignant B-CLL cells but also normal B cells, therefore cells that could induce antibody responses against the murine scFv-portion of the CAR would have been eliminated. CD19 CAR T cells will not be cleared by antibody-mediated responses and may therefore persist longer than CAR T cells directed against an antigenic structure on solid tumours. These details are important to keep in mind during further development of CAR T cell therapy for nonhaematopoietic tumours.

## Factors influencing the efficacy of TCR and CAR T cell therapy

Important issues to consider both for TCR and CAR T cell therapy are the gene transfer technology and the fact that the genetically engineered T cells must have optimal avidity for the tumour-associated antigen, which is determined by the affinity of the receptor and the number of receptors expressed on the surface of the engineered T cells. These cells must also be able to persist upon infusion and to expand *in vivo*. Furthermore, they need to be able to home to tumour sites and they must be safe (i.e. lack toxicity). These issues will be discussed in more detail below.

### Vectors and methods used for gene transfer to T cells

So far, most preclinical and clinical studies have used gamma-retroviral vectors for transfer of TCR and CAR genes into T cells. Retroviral vectors yield a high level of stable transgene expression through integration of the viral genome into a transcriptionally active but non controllable site of the host T cell genome. The efficiency of gene transfer using retroviral or lentiviral vectors shortens the time required for culturing T cells to reach clinically significant numbers. However, retroviral vectors can only efficiently transduce dividing cells. Therefore, target T cells must be pushed into the cell cycle by stimulation of the endogenous TCR to achieve a reasonable degree of transduction. Lentiviral vectors transduce most cell types without the requirement for recipient cells to undergo cell cycling. However, primary human lymphocytes tend to be fairly resistant to lentivirus transduction although, in principal, T cells can be transduced using lentiviral vectors with stimulating cytokines such as IL-2, IL-7 or IL-15 74. Pre activation of T cells before retroviral and lentiviral transduction yields much higher degrees of transduction and different approaches have been utilized including the use of the agonistic anti-CD3 antibody (OKT-3), CD3/CD28 magnetic beads and artificial APCs. These approaches may lead to preferential activation and expansion of either CD8^+^ or CD4^+^ T cell subsets and to yield different cytokine profiles. Furthermore, it has been argued that TCR activation impairs the half-life, repertoire and immune competence of the transduced T cells 75. Therefore, pre activation via the endogenous TCR for transduction might reduce the fitness of engineered T cells.

Concerns have been raised that transgene integration can lead to insertional mutagenesis and malignant transformation of the transduced T cells, as has been observed for retroviral gene transfer to haematopoietic stem cells 76. However, this risk is considered very low for fully mature lymphocytes, although rare events of T cell transformation have been detected when the retroviral vector carries the LMO-2 oncogene 77. The safety of using lentiviral vectors for TCR and CAR gene transfer is likely to be very high. A benign integration bias for lentiviral vectors without oncogenic selection has recently been demonstrated 78.

Non viral gene transfer of TCR and of CAR using a non integrating plasmid or *in vitro* transcribed mRNA have generally resulted in short-term transgene expression and fairly low efficacy 79–83. Adoptive transfer of T cells engineered using these approaches must be repeated multiple times for therapeutic effects. However, plasmid or mRNA transfer technologies represent attractive means of TCR and CAR gene transfer when the T-cell target antigen is not fully restricted to tumour cells and there are concerns about toxicity. The short half-life of such T cells *in vivo* would ensure safety. Another non viral transfer option is to use retrotransposon systems, such as the PiggyBac 84 or the Sleeping Beauty 85 systems. The TCR or CAR transgene in the transposon plasmid together with a transposase plasmid cause the TCR or CAR transgene integrate into the host T cell genome. Transposon systems are significantly more efficient for integration than normal DNA plasmids. However, at present, viral gene transfer seems to be the most feasible way to ensure stable long-term expression of TCRs or CARs in grafted cells. If methods of non viral gene modification improve in terms of gene transfer rates and stability of expression, they might become a safe and cheap alternative for clinical applications.

## *In vivo* persistence of genetically engineered T cells

The differentiation status of engineered T cells, alteration of the host environment into which the T cells are infused and the addition of supportive cytokines are all factors that are likely to influence *in vivo* persistence of adoptively transferred T cells.

### Pre selection of T cell subsets for gene transfer

At present, TCR- or CAR-engineered T cells infused into patients are usually generated from unselected CD4^+^ and CD8^+^ T cells from peripheral blood and will thus contain an unpredictable mixture of lymphocyte subsets. In some studies, CD8^+^ cytolytic T cells have been preselected for gene transfer. Resting CD8^+^ T cells exist as naïve (T_N_), central memory (T_CM_) and effector memory (T_EM_) populations, each with distinct phenotypic and functional characteristics 86. Riddell and colleagues elegantly showed that antigen-experienced CD8^+^ T_CM_ cells persisted longer than T_EM_ cells following adoptive transfer into primates. The authors used naturally isolated and *ex vivo*-expanded cytomegalovirus (CMV)-specific CD8^+^ T cells for comparison 87. Nick Restifo and colleagues developed the *Pmel-1* transgenic mouse model, in which more than 95% of all CD8^+^ T cells recognize an epitope from the murine gp100 melanoma-associated antigen, to study TCR gene transfer to mice with B16 melanoma 88. They found that T_N_ rather than T_CM_ cells gave rise to an effector population that mediated superior antitumour immunity upon adoptive transfer 89. These authors also identified a specific subset of CD8^+^ T cells with stem-like properties, termed stem cell memory T (T_SCM_) cells, which may be optimal for TCR gene transfer 90,91. It is important to note that, irrespective of the cell of origin, culture conditions used during and directly after gene transfer may affect the subsequent *in vivo* properties of T cells. Gene transfer is usually conducted after T cell activation and the cells are cultured in medium containing high doses of IL-2. These culture conditions induce T cell differentiation towards a late effector state. Bonini and colleagues have shown that costimulation and culture in the presence of IL-7 and/or IL-15 promote the expansion of gene-engineered T cells with an early differentiation phenotype and may allow greater expansion and prolonged *in vivo* persistence 92.

Another attractive approach is to select EBV-specific or CMV-specific T cells for TCR or CAR engineering 93. It is assumed that such TCR- or CAR-engineered T cells receive optimal and continuous costimulation through their native virus-specific TCR in patients with latent EBV or CMV infection and therefore survive longer and lead to long-lasting antitumour responses. However, the differentiation status and subset of T cells are also of outmost importance when selecting virus-specific T cells for gene transfer.

### Preconditioning of patients before T cell infusion

The role of lymphodepletion on the effectiveness of adoptive T cell transfer has been extensively studied in the *Pmel-1* mouse model with adoptive transfer of gp100-specific T cells into mice with established B16 melanoma tumours. It was found that increased intensity lymphodepletion prior to adoptive T cell transfer enhanced tumour treatment efficacy 94. Important contributing factors for lymphodepletion are depletion of T regulatory cells and homeostatic expansion of T_N_, T_CM_ and T_EM_ cells because of the accessibility of cytokines, which are crucial for homeostatic proliferation 95. Lymphodepletion has also shown benefit in clinical trials and increasing the intensity of the preconditioning regimen of TIL transfer to melanoma patients can increase response rates 96. It is noteworthy that all patients who have received adoptive transfer of TCR- or CAR-engineered T cells so far have been treated with various forms of chemotherapy for varying periods of time before entering the trials. Therefore, the preconditioning regimen may in the future be individualized and based on prior treatments. Furthermore, preconditioning may be less essential if highly persistent engineered T cells are transferred.

### Supportive cytokines for transferred T cells

Systemic administration of IL-2 is often used in clinical protocols to increase the persistence of transferred T cells 2,97. However, it is widely recognized that systemic IL-2 treatment causes significant toxicity, such as vascular leakage syndrome, which requires intensive care treatment, especially when high doses are used 98. Methods to avoid the need for systemic IL-2 administration include inserting cytokine genes or inducible cytokine genes into the transfer vector and thereby include local cytokine production in the transferred T cells that should persist *in vivo*. The *Pmel-1* mouse model was used to investigate whether or not insertion of IL-12 into gp100-specific CD8^+^ T cells was beneficial. It was found to increase the antitumour effect without the need for exogenous IL-2, although it did not increase overall survival 99. This mouse model was also used to evaluate the importance of T cell dosage, magnitude of *in vivo* antigen restimulation, the relative efficacy of T_CM_, T_EM_ and T_SCM_ subsets on the strength of tumour regression as well as the dose and type of clinically available γ(c) cytokines, including IL-2, IL-7, IL-15 and IL-21. T cell dose and differentiation status correlated strongly and significantly with the magnitude of tumour regression; however, there was little difference between the various cytokines. Furthermore, cytokine administration for more than 6 days did not improve outcome 100. These findings should guide the future design of clinical trials, although it should also be noted that results from mouse models can be misleading 101.

In the successful CD19 CAR T cell U-Penn trial, patients did not receive IL-2 infusion and yet the T cells expanded by up to 1 000-fold 4,5. It is likely that the T cells expanded in response either to homeostatic cytokines or to CD19 expressed on leukaemic target cells and/or normal B cells. Indeed, the kinetics of cytokine release in serum and bone marrow after the introduction of CD19 CAR T cells into patients correlated with a peak in CD19 CAR T cell numbers, which suggests that the decline in these cell numbers may be initiated when cellular targets expressing CD19 become limiting. This situation is preferable to a continuous non target cell-based expansion, which will cause lymphoproliferation upon infusion of CAR T cells.

### Homing of transferred T cells to tumour sites

Besides being able to persist *in vivo*, the genetically engineered T cells must efficiently traffic to the tumour sites and, once there, sustain their effectiveness in the presence of an array of immune evasion strategies used by the tumour cells. Homing may also be compromised by the loss of desired chemokine receptors during genetic modification and passage *in vitro*, or by the selection of T cells that are inherently unable to localize to certain tissues. Therefore, further genetic modification with relevant chemokine receptors may be advantageous. It has been shown that T cells engineered to express CXCR2 will preferentially traffic to melanomas 102, whereas T cells expressing CCR4 will traffic to Hodgkin's lymphoma 2009. Co-expression of a CAR targeting the CD30 antigen on Hodgkin's lymphoma with CCR4 enhanced antitumour activity *in vivo* in a xenograft model 2009.

### Long-term safety of genetically engineered T cells

Genetically engineered T cells may exert off-target or on-target/off-tumour toxicity. Moreover, they have the potential to last for a long time in the host and even expand in number. Therefore, any adverse toxicity may worsen over time. This is a particular concern when T cells are engineered to resist the physiological ‘off signals’ that are exploited by many cancers to subvert tumour immune recognition and effector function. Therefore, the ability to eradicate the transferred T cells, if needed, would be desirable.

A suicide gene can be included in the genetically engineered T cells along with the TCR or CAR transgene. The first and most widely used suicide gene is the herpes simplex virus thymidine kinase (HSV-tk), which can convert the nucleoside analogues ganciclovir and acyclovir to active compounds that efficiently kill HSV-tk-expressing cells 104. HSV-tk is potentially immunogenic, which can lead to unwanted immune-mediated destruction and thus loss of persistence of the genetically engineered T cells 105. More recently, an inducible system based on the use of a modified human caspase-9 fused with a human FK506-binding protein to allow conditional dimerization using a commercial dimerizing agent has been developed 107. Another approach, based on the fact that CD20-expressing cells can be eliminated by administration of rituximab, is to introduce CD20 as a non immunogenic suicide gene in the engineered T cells 108.

## Conclusions and future directions

Cancer therapy using genetically engineered T cells is still in its infancy and many approaches are being examined in parallel in small heterogenic groups of patients. The diversity of TCRs, CARs and vectors used in studies, the selection of various T cell subsets for gene transfer and the different preconditioning and supportive cytokine regimens available for patients are likely to lead to significant advances in the field of cancer immunotherapy. However, the diversity also means that it will be difficult to identify which particular aspects of a protocol are critical for its effectiveness. The relatively slow progress of T cell therapeutics into established drugs is also due to the low interest from the biotechnology industry to explore advanced biological therapeutic agents and invest in the field. However, due to the recent success with gene-engineered T cells and possibilities to commercialize gene transfer vectors, the potential of this upcoming therapy for cancer may soon be appreciated, leading to large randomized Phase III trials to prove the efficacy of these cells. To broaden patient access, it must be shown that genetically engineered T cells can be reproducibly manufactured to be clinically effective. Ultimately, it will be important to find out whether or not this novel and extremely promising form of therapy can deliver improvements in both progression-free survival and overall survival when compared with the standard of care.
